# Potential Mechanisms for the Ketamine-Induced Reduction of P3b Amplitudes

**DOI:** 10.3389/fnbeh.2018.00308

**Published:** 2018-12-11

**Authors:** Timm Rosburg, André Schmidt

**Affiliations:** ^1^Forensic Department, University Psychiatric Clinics Basel, Basel, Switzerland; ^2^Department of Psychiatry, University Psychiatric Clinics Basel, Basel, Switzerland

**Keywords:** Schizophrenia, event-related potentials, ketamine, task difficulty, working memory, N-methyl-D-aspartate, model psychosis

## Abstract

In specific dosages, the N-methyl-D-aspartate receptor (NMDA) antagonist ketamine can be used to model transient psychotic symptoms in healthy individuals that resemble those of schizophrenia. Ketamine administration also temporarily impairs cognitive functions, which can be studied by event-related potentials (ERPs). ERPs also allow dissecting what stages of information processing are affected by ketamine and what stages remain functional. For tasks requiring the differentiation of targets and non-targets, it has repeatedly been shown that ketamine administration in healthy individuals leads to decreased amplitudes of the ERP component P3b in response to target stimuli. However, it could be argued that this ketamine-induced P3b reduction is the consequence of an increased difficulty to differentiate targets from non-targets, primarily mediated by ketamine's psychotomimetic rather than pharmacological effects. The current review of ERP studies seeks to clarify the issue whether P3b effects of ketamine may indeed be explained as the consequence of an experienced increase in task difficulty or whether alternative mechanisms are perhaps more plausible. The review first summarizes the effects of task difficulty on ERP components related to intentional stimulus categorization (P3b), involuntary attention switches to distractors (P3a), as well as sensory processing (P1, N1). Secondly, the ERP effects of task difficulty are contrasted with those observed in ketamine studies in healthy individuals. Findings show that P3b amplitudes are consistently diminished by an increased task difficulty, as well as after ketamine administration. In contrast and as most important difference, increased task difficulty leads to increased P3a amplitudes to distractors presented in same modality as targets, whereas ketamine leads to reduced P3a amplitudes for such distractors. This dissociation indicates that the decreased P3b amplitudes after ketamine cannot be explained by a drug-induced increase in task difficulty. The conjoint reductions of P3a and P3b amplitudes instead suggest that working memory operations, in particular working memory updating are impaired after ketamine, which is in line with previous behavioral findings.

## Introduction

Various studies have administered subanesthetic doses of ketamine, an N-methyl-D-aspartate receptor (NMDA) antagonist, to model psychotic symptoms and psychosis-like experiences in healthy individuals. Krystal et al. (1994) were one of the first research groups who experimentally investigated these effects. They used a bolus of either 0.1 mg or 0.5 mg ketamine/kg body weight in a placebo-controlled, double-blind design. The bolus of 0.5 mg/kg temporarily evoked pronounced negative and positive symptoms and also resulted in a range of short-lasting cognitive impairments, whereas the bolus of 0.1 mg/kg had hardly any effect. Subsequent studies that aimed at eliciting psychotomimetic effects usually applied a single bolus of 0.1–0.3 mg/kg, followed by a continuous injection of 0.135–0.9 mg/kg per hour to ensure a lasting drug effect (Breier et al., 1997; Adler et al., 1998; van Berckel et al., 1998; Newcomer et al., 1999; Oranje et al., 2000, 2009; Umbricht et al., 2000; Vollenweider et al., 2000; Ahn et al., 2003; Boeijinga et al., 2007; Heekeren et al., 2008; Watson et al., 2009; Musso et al., 2011; Ebert et al., 2012; Gunduz-Bruce et al., 2012; Schmidt et al., 2012, 2013; Mathalon et al., 2014; Koychev et al., 2017).

**Figure 1:**
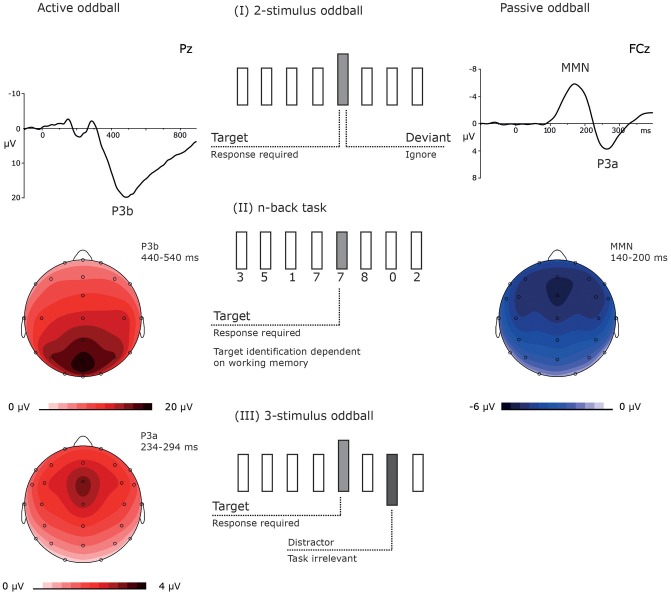
The figure shows three different types of oddball experiments. (I) The relatively common 2-stimulus oddball experiment contains two kinds of stimuli, one is frequently presented and the other less frequently, usually in < 20% of the trials. This kind of oddball can be used in active and passive conditions, primarily varying in their task instruction: In active oddball experiments, the rare stimulus is defined as target and requires an active response (e.g., a button press). Correctly identified targets elicit the P3b which maximal at centro-parietal electrodes and peaks between ~250 and 500 ms (left top: the ERP response at electrode Pz, left middle: the scalp distribution of the P3b; data were recorded in a visual oddball experiment; Rosburg et al., unpublished data). In passive oddball experiments, participants ignore the stimuli. The rare stimulus is defined as deviant and elicits the MMN, which is fronto-central negativity between 100 and 250 ms (right top: the difference potential between the ERP to duration deviants and the ERP to standard tones; right middle: the scalp distribution of the MMN; data from Rosburg et al. (2018). (II) The n-back represents a variant of the 2-stimulus oddball task, which is with increasing n more WM dependent. In the depicted 1-back condition, an immediately repeated stimulus is defined as target. This variant has hardly been used in passive conditions and elicits only weak MMN responses (e.g. Rosburg, 2004). (III) For active conditions also quite common 3-stimulus oddball experiments contain rare distractor stimuli in addition to targets and standards. These distractors do not require a behavioral response. Due to their perceptual salience and rare presentation, distractors elicit a P3a, which is also observed in passive oddball experiments in response to deviants (left bottom: topography of the P3a in a passive oddball experiment, data from Rosburg et al. (2018).

Some of the studies used event-related potentials (ERPs) in order to test whether such subanesthetic doses of ketamine lead to processing deficits in healthy individuals similar to those observed in patients with schizophrenia and to reveal the neurocognitive consequence of the drug administration (van Berckel et al., 1998; Oranje et al., 2000, 2009; Umbricht et al., 2000; Kreitschmann-Andermahr et al., 2001; Ahn et al., 2003; Boeijinga et al., 2007; Heekeren et al., 2008; Watson et al., 2009; Musso et al., 2011; Gunduz-Bruce et al., 2012; Schmidt et al., 2012, 2013; Mathalon et al., 2014; Koychev et al., 2017). In many of these ERP studies, ketamine effects were investigated in passive and active oddball experiments. The very same stimulation protocol might be used for both active and passive conditions, with only varying in instruction (Figure 1).

Two-stimulus oddball paradigms are quite commonly used. In this type of paradigm, there is one frequently and one rarely presented stimulus. In active oddball tasks, the rare stimulus is defined as target, which the participant has to identify by button press or another active response, whereas the second, frequently presented non-target stimulus requires no response (Duncan et al., 2009). The participant's identification of targets is associated with a large centro-parietal positive ERP component, the P3b, peaking ~250–500 ms after the stimulus target onset (Glossary). In passive 2-stimulus oddball experiments, participants are instructed to ignore all stimuli and often perform an active task in a second modality, like reading a book or watching a silent video, to make sure that the attention is directed away from the to-be-ignored stimuli. In passive 2-stimulus oddball experiments, the frequently presented stimulus is designated as standard and the rare stimulus as deviant. Auditory deviants in an otherwise uniform stimulation elicit the mismatch negativity (MMN), a fronto-central negativity peaking ~100 to 250 ms after stimulus onset, as first described by Näätänen et al. (1978). As the MMN is elicited even when individuals ignore the auditory stimulation, the component is considered to reflect pre-attentive memory-based change-detection mechanisms (Näätänen et al., 2011, Glossary).

Ketamine leads to an attenuation of the MMN amplitude (for review Rosburg and Kreitschmann-Andermahr, 2016), which parallels findings obtained in schizophrenia (for review Erickson et al., 2016). The similarity between the behavioral effects of NMDA receptor antagonists in healthy individuals and impairments/symptoms observed in schizophrenia (including the reduced MMN) provided support for the hypothesis that deficits in glutamatergic neurotransmission play a pivotal role in schizophrenia (Kantrowitz and Javitt, 2012). Besides the ketamine-induced deficit in pre-attentive deviance detection as indexed by the reduced MMN, ketamine administration in healthy subjects also diminishes the P3b amplitude, elicited by actively to-be-identified targets (e.g., Gunduz-Bruce et al., 2012; Mathalon et al., 2014). Similar to the impaired generation of the MMN, reduced P3b amplitudes, in particular to auditory stimuli, represent another consistent ERP finding in schizophrenia research and are considered as a potential biomarker for schizophrenia (Bramon et al., 2004; Thibaut et al., 2015; Earls et al., 2016).

The P3b is considered to reflect the neural correlate of context updating in working memory (Polich, 2007). According to this account, perceived stimuli are categorized as “target” or “non-targets”: The neural representation of the stimulus environment remains unaffected by the presentation of non-targets, whereas the identification of targets results in the allocation of attentional resources and an updated neural representation of the stimulus environment (Polich, 2007). The P3b as an endogenous component depends on the individual's perception of stimuli as targets and their reaction onto them and is, therefore, dependent on the participant's willingness and ability to perform the requested task. Given this, it is not fully clear which cognitive processes in active oddball tasks are disrupted by ketamine. Is already the perception of stimuli impaired, becomes the differentiation of targets and non-targets more difficult, or are working memory functions compromised by the drug? All factors could in principle result in reduced P3b amplitudes.

Schwertner et al. (2018) recently reviewed previous studies investigating the impact of ketamine on the P3b in response to targets, the P3a in response to distractors, as well as on ERP components associated with sensory processing such as the auditory and visual P1 and N1 (Glossary). The authors reported that ketamine administration in healthy individuals was associated with decreases in certain ERP component amplitudes such as the P3b, whereas early ERP components such as the P1 and N1 were unaffected or even increased by ketamine. They suggest that “ketamine may alter the perceived salience of different categories of stimuli” and may lead to changes in cognitive resources dedicated in the interpretation of stimuli (Schwertner et al., 2018, p. 11). As an example, they line out that in oddball experiments the P3b amplitude to targets decreases and the P3a amplitude to distractors increases when the discriminability of standards and targets decreases and thus target identification becomes more difficult (Hagen et al., 2006). They then suggest that “If ketamine alters the perceived relationship between the target and standard stimuli in the oddball task, it could potentially account for the P300 reductions and N100/P100 increases reported in most studies.” (p. 11).

Following the argumentation of Schwertner et al. (2018), the decreased discriminability of standards and targets represents a direct drug effect of ketamine. This would suggest that ketamine directly impairs cognitive functions supporting the target detection due to its pharmacological properties. However, alternatively, individuals might experience greater difficulties in identifying targets due to the psychotomimetic effects of ketamine, e.g., participants might be distracted from the target identification due to drug-induced perceptual alterations (Krystal et al., 1994). In this case, one would argue that the ketamine effects reflected in the P3b amplitude reductions are more indirectly linked to the pharmacological properties of ketamine.

The current review aims at evaluating empirical evidence whether the ketamine-induced ERP alterations in active oddball experiments can be explained by an increased task difficulty, either directly related to ketamine's pharmacological properties or more indirectly to its psychotomimetic effects, or whether alternative mechanisms are more plausible. For this purpose, we initially review findings on the impact of increased task difficulty on the P3b and other ERP components. Then, we inspect ERP studies that have investigated the effects of ketamine in order to compare the observed ketamine effects with those induced by increased task difficulty. This comparison includes the ERP components P3a and P3b, P1, N1, and Processing Negativity (PN), as well as the task accuracy.

## Methods

In the first result section, we present a selective review of previous studies showing how the ERP components P3a and P3b, P1, N1, and PN of healthy individuals are modulated by an increased task difficulty in active oddball tasks. Task difficulty in oddball experiments can be modulated by a range of factors (for review Kok, 2001), whereby here we focused on the effects of decreasing the discriminability of targets vs. non-targets, introducing a secondary task, increasing the task load of either the primary or secondary task, or by presenting distractor stimuli in the same or a different modality than targets. From our point of view, these experimental modulations mimic the hypothesized direct or indirect effects of ketamine best: Ketamine might decrease the ability to discriminate targets and standards, it might distract from the target detection task, or alternatively, it might diminish attentional and cognitive resources required for performing the target detection task. Moreover, the mentioned experimental variations of increasing the task difficulty correspond to tasks used in ketamine studies.

In the second result section, we review how the ERP components P3a and P3b, P1, N1, and PN of healthy individuals are modulated by subanesthetic doses of ketamine. The review of ketamine effects is widely based on the same literature as the very recent review of Schwertner et al. (2018). However, studies were only included when the administered ketamine dose was large enough to evoke psychotomimetic effects in healthy participants. As suggested by the studies of Krystal et al. (1994) and Newcomer et al. (1999), it requires a bolus of more than 0.1 mg/kg or a bolus more than 0.0243 mg/kg followed by continuous injection of more than 0.012 mg/kg bodyweight per hour to observe such effects. We included ERP studies that were clearly above these thresholds (single bolus of 0.1–0.3 mg/kg, eventually followed by a continuous injection of 0.135–0.9 mg/kg per hour, for a detailed listing of the used doses see Table 2 in Schwertner et al., 2018). Consequently, we excluded the studies of Murck et al. (2006) and Knott et al. (2011), administering doses clearly below these thresholds (Murck et al., 2006: no bolus and continuous injection of approximately 0.001 mg/kg per hour; Knott et al., 2011: single bolus of 0.04 mg/kg). Knott et al. (2011) themselves qualified their study dose as “subperceptual.” In variation from Schwertner et al. (2018), we did not consider the study of Umbricht et al. (2002) in this review, because the relevant data were the same as reported by Umbricht et al. (2000). From the study of Kort et al. (2017), we considered only data from the passive listening condition because the N1 suppression by speech production includes other processes than the sensory processing of the auditory stimuli. In contrast to Schwertner et al. (2018), we additionally included the prepulse inhibition study of Boeijinga et al. (2007), but only considered the pulse alone condition.

## Results

### Impact of Task Difficulty on ERPs

An increased task difficulty is presumed to be reflected in poorer task accuracy and slower reaction times. Thus, experimental manipulations that seek to increase the task difficulty should always result in such behavioral changes. Indeed, most experimental manipulations successfully modulated the task difficulty as reflected in the two behavioral markers (Tables 1A–D, right columns).

**Table 1A:** Decreased discriminability of standards and targets/task cues in active oddballs.

**Study**	**Task**	**Task difficulty increased by**	**P1/N1**	**PN**	**P3a**	**P3b**	**Accuracy**	**RTs**
Fitzgerald and Picton, 1983	3-stimulus auditory oddball	Decreased discriminability of standards and targets	↓ (N1)	—	—	↓	↓	↓
Fitzgerald and Picton, 1984	2-stimulus auditory oddball	Decreased discriminability of standards and targets	↓ (N1)	—	—	↓	↓	—
Polich, 1987	2-stimulus auditory oddball	Decreased discriminability of standards and targets	—	—	—	↓	—	—
Scheffers et al., 1991	Visual choice reaction times task	Decreased discriminability of task cues	—	—	—	↓	↓	↑
Alho et al., 1992	3-stimulus auditory oddball	Decreased discriminability of standards and targets and secondary task	—	↑	—	↓	↓	↑
	3-stimulus visual oddball	Decreased discriminability of standards and targets and secondary task	—	↑	—	↓	↓	↑
Mehaffey et al., 1993	2-stimulus visual oddball	Decreased discriminability of standards and targets	—	—	—	↓	—	—
Katayama and Polich, 1998	3-stimulus auditory oddball	Decreased discriminability of standards and targets	—	—	↑ distractors same modality	↓	↓	↑
Comerchero and Polich, 1999	3-stimulus auditory or visual oddball task	Decreased discriminability of standards and targets	—	—	↑ distractors same modality	↓	↓	↑
Handy and Mangun, 2000	Visual choice reaction times task	Decreased discriminability of task cues	↑ (P1)	—	—	—	↓	↑
Demiralp et al., 2001	3-stimulus visual oddball	Decreased discriminability of standards and targets	—	—	↑ distractors same modality	↓	↓	↑
Polich and Comerchero, 2003	3-stimulus visual oddball	Decreased discriminability of standards and targets	—	—	↑ distractors same modality	↓	↓	↑
Combs and Polich, 2006	3-stimulus auditory oddball	Decreased discriminability of standards and targets	—	—	↑ distractors same modality	↓	↓	↑
Hagen et al., 2006	3-stimulus visual oddball	Decreased discriminability of standards and targets	—	—	↑ distractors same modality	↓	↓	↑
Muller-Gass et al., 2006 Exp. 1/ I	2-stimulus visual oddball	Decreased discriminability of visual standards and targets	—	↑	—	↓	↓	↑
Muller-Gass et al., 2006 Exp. 2/ I	2-stimulus visual oddball	Decreased discriminability of visual standards and targets	—	↑	—	↓	↓	↑
Sawaki and Katayama, 2006	3-stimulus visual oddball	Decreased discriminability of standards and targets	—	—	↑ distractors same modality	↓	↓	↑
Gaál et al., 2007	2-stimulus auditory oddball	Decreased discriminability of standards and targets	—	—	—	↓	Ø	↑
Matthews et al., 2009	3-stimulus visual oddball	Decreased discriminability of visual standards and targets and secondary task	—	—	↑ distractors same modality	↓	↓	↑
Frank et al., 2012	3-stimulus auditory oddball	Decreased discriminability of standards and targets	—	—	↑ distractors same modality	↓	↓	↑
Yurgil and Golob, 2013	3-stimulus auditory oddball	Decreased discriminability of standards and targets	↑ (N1 targets)	—	↓ white noise as distractors	↓	↓	↑
Lange and Schnuerch, 2014	3-stimulus auditory oddball	Decreased discriminability of standards and targets	↑ (N1)	—	—	Ø	↓	↑
Sugimoto and Katayama, 2017	3-stimulus visual or visual-auditory oddball	Decreased discriminability of standards and targets	Ø (auditory N1)	—	↑ distractors in both modalities	↓	↓	↑

The table summarizes the previously reported effects of decreased discriminability of standards and targets (or other task cues) in active oddballs on the N1/P1, PN, P3a, and P3b amplitudes, as well as the resulting behavioral effects (accuracy and reactions times, RTs). Decreased discriminability should result in an increased task difficulty and, thus, should be reflected in decreased task accuracy (↓) and increased RTs (↑). For the ERP amplitudes, downward tilted arrows (↓) indicate decreased amplitudes, upward tilted arrows (↑) increased amplitudes, with decreased discriminability of standards and targets. Absent effects are indicated by “Ø” and unreported effects by “—“

**Table 1B:** Increased primary task difficulty in passive oddball experiments.

**Study**	**Stimulation**	**Primary task**	**P1/N1**	**PN**	**P3a**	**P3b**	**Accuracy**	**RTs**
Harmony et al., 2000	2-stimulus auditory oddball	Visual task varied in difficulty (increased WM load)	Ø	—	↓ (deviants)	—	—	—
Berti and Schröger, 2003	2-stimulus auditory oddball	Auditory task varied in difficulty (increased WM load)	↓ (N1)	—	↓ (deviants)	—	↓	↑
Restuccia et al., 2005	2-stimulus auditory oddball	Visual task varied in difficulty	↓ (N1)	—	↓ (deviants)	—	—	—
Yucel et al., 2005	3-stimulus auditory oddball	Visuo-motor tracking task varied in difficulty	Ø (N1)	—	Ø (deviants) ↓ (novels)	—	↓	—
Muller-Gass et al., 2006 Exp. 1 / I	3-stimulus auditory oddball	Visual discrimination task (oddball)	—	—	Ø	—	↓	↑
Muller-Gass et al., 2006 Exp. 2 / I	3-stimulus auditory oddball	Visual discrimination task (oddball)	—	—	↓ (intensity deviants)	—	↓	↑
Zhang et al., 2006	2-stimulus auditory oddball	Visual tracking task varied in difficulty	Ø (N1 standards) ↑ (N1 deviants)	—	↓ (deviants)	—	↓	—
Allison and Polich, 2008	1-stimulus auditory oddball	Visual task (video game), varied in difficulty	↓ (N1)	—	↓ (rare)	—	↓	—
SanMiguel et al., 2008	2-stimulus auditory oddball	Visual task (CPT) varied in difficulty	—	↑	↓ (novels)	—	↓	↑
Sculthorpe et al., 2008	2-stimulus auditory oddball	Visual tracking task varied in difficulty	—	—	↓ (deviants)	—	↓	↑
Wronka et al., 2008	3-stimulus auditory oddball	Passive listening during secondary task visual vs. active target identification	—	—	↓ (deviants)	—	—	—
Lv et al., 2010	2-stimulus auditory oddball	Visual task varied in difficulty	—	—	↓ (novels)	—	↓	↑
Miller et al., 2011	1-stimulus auditory oddball	Visual task (tetris) with sound, varied in difficulty	↓	—	↓ (rare)	—	↓	—
Sugimoto and Katayama, 2013	2-stimulus somatosensory oddball	Visual task (video game) varied in difficulty	—	—	Ø (deviants)	—	—	—
Takeda et al., 2014	2-stimulus auditory oddball	Poor vs. enriched visual virtual reality environment	Ø	—	↓ (deviants)	—	—	—
Dyke et al., 2015	1-stimulus auditory oddball	Visual task (tetris), varied in difficulty	Ø	—	↓ (rare)	—	↓	—
Molloy et al., 2015	4-stimulus auditory oddball	Visual task varied in difficulty	↓ (auditory) ↑ (visual)	—	↓ (rare)	—	↓	↑
Simon et al., 2016	3-stimulus auditory oddball	Increased WM load, secondary auditory oddball task	↓ (N1)	—	—	—	↓	↑
Morlet et al., 2017	2-stimulus auditory oddball	Imagery vs. mind-wandering	—	—	↓ (deviants)	—	—	—
Tusch et al., 2017	3-stimulus auditory oddball	Increased WM load, secondary auditory oddball task	—	—	↓	—	↓	↑

*The table summarizes the previously reported effects of an increased primary task difficulty on the N1/P1, PN, and P3a amplitudes in passive oddball paradigms, as well as the resulting behavioral effects (accuracy and RTs) in the primary task. In passive oddball paradigms, the deviant is defined as task-irrelevant and does not require a behavioral response by the participant. Given this, there is no target-related P3b in the ERPs. For the purpose of the current review, the passive oddball paradigm is defined as secondary task (“stimulation”). For the meaning of the used symbols please see Table 1A*.

**Table 1C:** Dual task conditions.

**Study**	**Task**	**Task difficulty increased by**	**P1/N1**	**PN**	**P3a**	**P3b**	**Accuracy**	**RTs**
Isreal et al., 1980	2-stimulus auditory oddball	Visual monitoring task with varying difficulty	—	—	—	↓	—	↑
Wickens et al., 1983	2-stimulus visual or auditory oddball	Visual tracking task	—	—	—	↓	—	—
Alho et al., 1992	3-stimulus auditory oddball	Visual stimulation and decreased discriminability of standards and targets	↑	—	—	↓	↓	↑
	3-stimulus visual oddball	Auditory stimulation and decreased discriminability of standards and targets	↑	—	—	↓	↓	↑
Kramer et al., 1995	3-stimulus auditory oddball	Visual task (radar monitoring) varied in difficulty	↓	—	↓	↓	↓	↑
Schubert et al., 1998	3-stimulus auditory oddball	Motor task varied in difficulty	Ø	—	Ø	↓	Ø	↑
Ullsperger et al., 2001	3-stimulus auditory oddball	Visual task (single vs. dual)	↓	—	↓	↓	↓	↑
Matthews et al., 2006	3-stimulus visual oddball	Motor task	—	—	↓	↓	↓	↑
Muller-Gass et al., 2006 Exp. 1/ II	2-stimulus visual oddball	Focused vs. divided attention	—	↑	—	↓	↓	↑
	3-stimulus auditory oddball	Secondary task varied in difficulty	—	↑	—	Ø	Ø	Ø
Muller-Gass et al., 2006 Exp. 2/ II	2-stimulus visual oddball	Focused vs. divided attention	Ø	—	—	↓	↓ (visual)	↑
	3-stimulus auditory oddball	Secondary task varied in difficulty	—	↑	—	Ø	↓	Ø
Allison and Polich, 2008	1-stimulus auditory oddball	Secondary task (video game), varied in difficulty	—		—	↓	↓	—
Pratt et al., 2011	Flanker task	Visual working memory task, varied in task load	↓		—	↓	↓	↑
Ries et al., 2016	Visual target detection	Auditory WM task with increasing load	↓		—	↓	↓ (auditory) Ø (visual)	↑

*The table summarizes the previously reported effects of dual task conditions in active oddballs on the N1/P1, PN, P3a, and P3b amplitudes, as well as the resulting behavioral effects (accuracy and RTs). For the meaning of the used symbols please see Table 1A*.

**Table 1D:** Increased working memory (WM) load in active tasks.

**Study**	**Task**	**Task difficulty increased by**	**P1/N1**	**PN**	**P3a**	**P3b**	**Accuracy**	**RTs**
Mecklinger et al., 1992	Visual WM task	Increased WM load	—	—	—	↓	↓	↑
Lorist et al., 1994	Visual WM task	Increased WM load	—	—	Ø (irrelevant visual cues)	↓	↓	↑
McEvoy et al., 2001	Visual WM task	Increased WM load	—	—	—	Ø	↓	↑
Watter et al., 2001	Visual WM task	Increased WM load	—	—	—	↓	↓	↑
Wintink et al., 2001	Visual WM task	Increased WM load	—	—	—	↓	—	—
SanMiguel et al., 2008	Visual WM task	Increased WM load	—	↑	—	↓	↓	↑
Chen and Mitra, 2009	Visual WM task	Increased WM load	—	—	—	↓	↓	↑
Ruhnau et al., 2010	Visual WM task	Increased WM load, 2-stimulus auditory oddball (passive); auditory stimuli preceded visual stimuli by 600 ms	—	—	↑ (auditory)	↓	↓	↑
Daffner et al., 2011	Visual WM task	Increased WM load	—	—	—	↓/↑depending on performance	↓	↑
Han et al., 2013	Visual WM task	Increased WM load	↓	—	—	↓	↓	↑
Scharinger et al., 2015	Visual WM task	Increased WM load and stimulus incongruence	—	—	—	↓	↓	↑
Causse et al., 2016	Visual tracking task	Increased WM load	—	—	—	↓	↓	—
Simon et al., 2016	Visual WM task	Increased WM load, secondary auditory oddball task	—	—	—	↓	↓	↑
Tusch et al., 2016	Visual WM task	Increased WM load	—	—	—	↓	↓	↑
Jiang and Rau, 2017	Visual WM task	Increased WM load	—	—	—	↓	↓	↑
Scharinger et al., 2017	Visual WM task	Increased WM load	—	—	—	↓	↓	↑
Tusch et al., 2017	Visual WM task	Increased WM load (multiple targets)	—	—	—	↓	↓	↑
Zhang et al., 2017	Visual WM task	Increased WM load	—	—	—	↓	↓	↑
Gajewski and Falkenstein, 2018	Visual WM task	Increased WM load	—	—	↑	↓	↓	↑

*The table summarizes the previously reported effects of an increased WM load in active tasks on the N1/P1, PN, P3a, and P3b, as well as on the task accuracy and RTs. For the meaning of the used symbols please see Table 1A*.

#### P1/N1/PN

The impact of task difficulty on ERP components related to sensory processing, such as the auditory N1 or the visual P1, is not easily assessable. One factor contributing to this challenge is that both ERP components are modulated by physical characteristics of the auditory and visual stimuli (for review Näätänen and Picton, 1987; Tobimatsu and Celesia, 2006). In studies varying the perceptual task difficulty (or perceptual load), the physical characteristics of easy and difficult targets differed. A higher perceptual load is usually achieved by lower target-standard discriminability (Table 1A). In other words, difficult targets are more similar to standards than easy targets. Fitzgerald and Picton (1983) used a standard tone of 1,000 Hz, a difficult target stimulus of 1,050 Hz, and an easy target stimulus of 1,500 Hz. In this study, easy targets elicited a significantly larger N1 than difficult targets. For auditory stimulation, it is, however, a well-established finding that greater frequency separation results in less stimulus-specific adaptation (SSA) and, in consequence, larger N1 amplitudes (Butler, 1968; Ulanovsky et al., 2003; Briley and Krumbholz, 2013). Given these findings, the larger N1 amplitudes for easy targets as observed by Fitzgerald and Picton (1983, 1984) were likely caused by a lessened impact of SSA and unrelated to task difficulty.

Some of the studies varying the perceptual load included a passive oddball condition. This allowed investigating whether the attentional modulation differed between test conditions with varying perceptual task difficulty. Directed attention increases both the auditory N1 and visual P1 amplitudes (N1: Hillyard et al., 1973; Näätänen et al., 1981; Hackley et al., 1990; Woldorff and Hillyard, 1991; Alho et al., 1992; P1: Hopfinger and Mangun, 1998; Taylor, 2002). For auditory stimulation, this attention effect is usually quantified by calculating the difference potentials between the auditory evoked potentials (AEPs) to attended stimuli and non-attended stimuli. The increased negativity to attended auditory stimuli is labeled as PN (or Negativity difference, Nd) (Näätänen, 1982, 1990; Woldorff and Hillyard, 1991). Alho et al. (1992) and Muller-Gass et al. (2006) showed that increased perceptual task difficulty results in an increased PN (Table 1A). Other recent studies suggest that more difficult tasks require more attentional resources, leading to larger P1 and N1 responses (Handy and Mangun, 2000; Lange and Schnuerch, 2014). In line with that, Schwent et al. (1976) showed that the attentional modulation of the auditory N1 is larger when sound events are harder to discriminate. Finally, increased levels of effort have been associated with increased N1 responses (Wilkinson and Morlock, 1967; Mulert et al., 2005, 2008). Thus, for active oddball paradigms, there is some evidence that increased perceptual load leads to increased P1 and N1 amplitudes.

Findings from passive oddball experiments with a primary task tend to show the opposite effect. In such experiments, participants are instructed to pay full attention to the primary task and to ignore the auditory stimulation. Under such conditions, more difficult primary tasks should be associated with decreased N1 amplitudes to the to-be-ignored auditory stimuli because with increasing task difficulty more attentional resources need to be allocated to the primary task. Indeed, reduced N1 amplitudes in passive oddball experiments have been reported for more demanding primary tasks (Berti and Schröger, 2003; Restuccia et al., 2005; Allison and Polich, 2008; Miller et al., 2011; Simon et al., 2016; Table 1B). With a more demanding primary visual task, Molloy et al. (2015) showed not only a decrease of the N1 amplitude to the to-be-ignored auditory stimuli, but interestingly also an increase of the visual P1. However, some studies observed no modulation of the auditory N1 with an increased primary task difficulty (Harmony et al., 2000; Yucel et al., 2005; Takeda et al., 2014; Dyke et al., 2015) and few studies revealed even increased N1 amplitudes (Zhang et al., 2006; SanMiguel et al., 2008).

In dual task conditions, findings are rather mixed, with some studies reporting decreased and others reporting increased P1/N1 amplitudes (Table 1C). Most studies did not instruct the participants to prioritize one task over the other (see Matthews et al., 2006 for an exception). Thus, increased task difficulty might lead to increased P1/N1 amplitudes in one task at the expense of decreased P1/N1 amplitudes in the second task, similarly as observed by Molloy et al. (2015).

#### P3a/P3b

The P3b is usually elicited in active oddball paradigms, using one frequent standard stimulus and one rare target stimulus, which requires a button press or needs to be counted (see Polich, 2007 for review). Already early studies suggested that increased task difficulty results in decreased P3b amplitudes (e.g., Isreal et al., 1980; Fitzgerald and Picton, 1983; Wickens et al., 1983) and this finding has repeatedly been replicated across different settings: Reduced P3b amplitudes with increased task difficulty have consistently been shown for studies varying the perceptual load (Table 1A), dual task conditions (Table 1C), and studies varying the working memory (WM) load (Table 1D).

The P3a is elicited by presenting rare events that considerably vary from the standards but do not require a response. These task-irrelevant events can distract individuals from the processing of task-relevant stimuli (Parmentier, 2014). When rare task-irrelevant events are presented within the same modality as the targets, the P3a amplitude (to these distractors) increases with increasing task difficulty (Katayama and Polich, 1998; Comerchero and Polich, 1999; Demiralp et al., 2001; Polich and Comerchero, 2003; Combs and Polich, 2006; Hagen et al., 2006; Matthews et al., 2009; Frank et al., 2012; Sugimoto and Katayama, 2017; Table 1A). This finding suggests that the processing of such distractors is more strongly engaged in harder target detection tasks. In contrast, when rare, task-irrelevant auditory stimuli had to be ignored and were presented during a primary visual task, the (early) P3a amplitude to these auditory stimuli decreased with increasing difficulty in the primary task or as compared to conditions without a visual task (Harmony et al., 2000; Restuccia et al., 2005; Yucel et al., 2005; Muller-Gass et al., 2006; Zhang et al., 2006; Allison and Polich, 2008; SanMiguel et al., 2008; Sculthorpe et al., 2008; Lv et al., 2010; Miller et al., 2011; Dyke et al., 2015; Molloy et al., 2015; Morlet et al., 2017; Tusch et al., 2017, Table 1B). Even though the P3a in passive oddball tasks is considered to reflect an involuntary attention switch in response to sound deviance (Näätänen, 1990; Escera et al., 2000), the P3a is stronger when participants attend the auditory stimuli (Wronka et al., 2008). Thus, the decreased P3a amplitudes in passive oddball experiments with increasing difficulty in the primary task suggest that more attentional resources were dedicated to the primary task and less resources to listening to the auditory stimulation. In dual task conditions, demanding visual, and motor tasks might also lead to a reduction of the P3a to attended auditory stimuli (Kramer et al., 1995; Ullsperger et al., 2001; Matthews et al., 2006, Table 1C). Thus, with the greater need to focus attention on one modality, the processing of distractors in the second modality is suppressed.

At first glance, some findings appear to conflict with the described pattern. In a visual oddball task, Sugimoto and Katayama (2017) observed an increased P3a not only to visual distractors, what would be in line with other findings, but also to auditory distractors (Table 1A). What could explain an attentional spill-over from one modality to the other, which is constantly absent in passive oddball experiments? In their experimental set-up, Sugimoto and Katayama (2017) replaced the visual distractor by an auditory distractor. Thus, the distracting event was not only characterized by a rare sound, but also by an absent visual stimulation. Therefore, the “auditory” distractor in this study actually represented an audio-visual distractor. Similarly, Ruhnau et al. (2010) observed an increased P3a to auditory cues in a visual WM task with increasing task load (Table 1D), again indicating some attentional spill-over from one modality to the other. In their study, the auditory cues always preceded the visual stimuli by 600 ms. In other words, the auditory cues informed about the timing of the to-be-attended visual stimuli and were therefore likely not ignored.

### Impact of Ketamine on ERPs

#### P1/N1/PN

In their recent review of ketamine effects on ERPs, Schwertner et al. (2018) summarized that the P1 and N1 amplitudes remain stable after ketamine administration or are even increased. However, it is not clear why Schwertner et al. (2018) neglected findings demonstrating decreased P1 and N1 amplitudes after ketamine, as reported by Boeijinga et al. (2007) for the neuromagnetic P1 and N1 to auditory stimuli (pulse only), Watson et al. (2009) for visual N1 to standards, or Schmidt et al. (2013) for the N170 in response to emotional face stimuli. The findings on alterations of the P1/N1 after ketamine are indeed rather mixed, with most studies reporting no drug effect on the P1/N1 and with the same number of studies reporting either decreased or increased P1/N1 amplitudes after ketamine (Table 2).

**Table 2:** Effects of ketamine on ERP amplitudes and task performance.

**Study**	**Task**	**P1**	**N1**	**PN**	**P3a**	**P3b**	**Hit rate**	**RTs**
**ACTIVE TASKS**								
Oranje et al., 2000	Dichotic listening	—	↑ (deviants)	↓	—	↓	Ø	Ø
Oranje et al., 2009	Dichotic listening	—	—	↓	—	↓	Ø	Ø
Watson et al., 2009	3-stimulus visual oddball	—	↓ (standards)	—	↓^+++^	↓	Ø	↓
Musso et al., 2011	2-stimulus visual oddball	—	—	—	—	↓	↓	Ø
Gunduz-Bruce et al., 2012	3-stimulus auditory oddball	—	—	—	↓	↓	Ø	Ø
Mathalon et al., 2014	3-stimulus auditory oddball	—	—	—	↓	↓	—	—
Ahn et al., 2003	Visual working memory	—	—	—	—	↓	↓^+^	—
Koychev et al., 2017	Visual working memory	↑	—	—	—	Ø	↓^++^	—
Schmidt et al., 2013	Facial affect discrimination	Ø	↓	—	—	—	↓	—
**PASSIVE TASKS**								
van Berckel et al., 1998	Auditory sensory gating	Ø	—	—	—	—	—	—
Oranje et al., 2002	Auditory sensory gating	Ø	—	—	—	—	—	—
Boeijinga et al., 2007	Prepulse inhibition	↓	↓	—	—	—	—	—
Umbricht et al., 2000	Passive auditory oddball	—	↑ (standards)	—	—	—	—	—
Kreitschmann-Andermahr et al., 2001	Passive auditory oddball	—	Ø (standards)	—	—	—	—	—
Heekeren et al., 2008	Passive auditory oddball	—	Ø (standards)	—	—	—	—	—
Kort et al., 2017	Passive listening	—	Ø	—	—	—	—	—

*The table lists the reviewed ketamine studies and summarizes the effects of ketamine administration on ERP amplitudes, as well as on task accuracy and reaction times. The studies are ordered according to the used task and publication year. Reductions of the P3a and P3b amplitudes after ketamine represent a relatively consistent finding across studies. In all studies reporting P3a effects, the distractors were presented in the same modality as targets*.

+ Marginally significant;

++ significant interaction between drug and working memory (WM) load; after ketamine task accuracy decreased more with increasing task load than after placebo;

+++ *only at Pz. For the meaning of the used symbols please see Table 1A*.

Watson et al. (2009) analyzed their data also in respect whether sensory deficits induced by ketamine (as reflected in the diminished N1 amplitude to standards) could contribute to the reduced P3a/P3b after ketamine. However, they did not find any associations between these effects. Moreover, Schmidt et al. (2013) did not reveal any association between the drug-induced worsened performance in emotional face recognition and the likewise reduced N170 response to these stimuli. Both findings argue against a link between the experienced task difficulty and ketamine-related P1/N1 reductions.

Two studies investigated the effects of ketamine on the PN and revealed decreased PN amplitudes after ketamine (Oranje et al., 2000, 2009). As outlined above, increased task difficulty to attended stimuli is associated with increased PN amplitudes (Schwent et al., 1976; Alho et al., 1992; Muller-Gass et al., 2006), reflecting the increased allocation of attentional resources under such conditions. Diminished PN amplitudes after ketamine would thus not conform to the idea that participants experienced the task as more difficult after ketamine. Conversely, increased task difficulty in a primary task is associated with decreased N1 amplitudes to ignored stimuli (Alho et al., 1992; Restuccia et al., 2005; Allison and Polich, 2008; Miller et al., 2011; Molloy et al., 2015; Simon et al., 2016), whereas the N1 in passive listening tasks was found to be unaffected (Kreitschmann-Andermahr et al., 2001; Heekeren et al., 2008; Kort et al., 2017) or increased (Umbricht et al., 2000) after ketamine.

Nevertheless, the rather mixed findings regarding the ketamine effects on the auditory and visual P1/N1 suggest that this field requires further research in order to clarify whether ketamine has or has not systematic effects on early sensory processing. On the basis of the available information, findings in one or the other direction can hardly be invalidated, although maybe with one exception: Most studies reporting significant effects on the P1/N1 used a cross-over double-blind design. In contrast, Koychev et al. (2017) investigated the effects of workload on working memory and ERPs after either ketamine or placebo administration in two distinct samples and did not include a pre-infusion baseline recording either. A comparison between the P1 data of Koychev et al. (2017) and a second study of this group using the same experimental design suggests that the P1 in the ketamine group was in the range of other healthy controls, whereas the P1 in the placebo group was comparably small (Koychev et al., 2010). With other words, the reported P1 effect of Koychev et al. (2017) was likely not due to ketamine, but to sample characteristics.

Aside from the mixed findings in human studies, more studies on the effects of ketamine on sensory ERPs are also required because of discrepancies between human and animal studies. Two studies on monkeys provide a relatively consistent pattern of results and suggest that the effects of NMDA receptor antagonists on the P1/N1 to auditory stimuli crucially depend on the interstimulus interval (ISI), with reduced P1/N1 amplitudes after ketamine administration becoming more apparent at long ISIs (Javitt et al., 2000; Holliday et al., 2017). These animal data might provide an explanation why some human studies did not observe a modulation of the P1/N1 after ketamine, as these studies used short ISIs (Kreitschmann-Andermahr et al., 2001; Heekeren et al., 2008; Kort et al., 2017). However, both the absence of decreased auditory P1 amplitudes after ketamine at long ISIs (van Berckel et al., 1998; Oranje et al., 2002) as well as the observation of increased auditory N1 amplitudes after ketamine (both at short and long ISIs) (Oranje et al., 2000; Umbricht et al., 2000) conflict with the animal data and cannot be attributed to methodological aspects.

#### P3a/ P3b/ Task Accuracy

Effects of ketamine on the P3b were investigated in 8 studies, with 4 studies using an oddball paradigm (Watson et al., 2009; Musso et al., 2011; Gunduz-Bruce et al., 2012; Mathalon et al., 2014), as well as 2 studies each using a working memory paradigm (Ahn et al., 2003; Koychev et al., 2017) and dichotic listening task (Oranje et al., 2000, 2009). Seven of these studies revealed decreased P3b amplitudes after ketamine (Table 2). The decreased P3b amplitudes after ketamine exposure appears to indicate that participants experienced the task as more difficult, given the plethora of studies showing that increased task difficulty is associated with decreased P3b amplitudes (e.g., Isreal et al., 1980; Fitzgerald and Picton, 1983, 1984; Wickens et al., 1983; Polich, 1987; Alho et al., 1992; Muller-Gass et al., 2006; Allison and Polich, 2008; Ruhnau et al., 2010; Pratt et al., 2011; Ries et al., 2016; Scharinger et al., 2017).

The impact of ketamine on task performance was quantified in 7 of these studies. 3 studies (including both WM task studies) reported diminished task accuracy after ketamine (Ahn et al., 2003; Musso et al., 2011; Koychev et al., 2017), whereas 4 studies observed no such effect (Oranje et al., 2000, 2009; Watson et al., 2009; Gunduz-Bruce et al., 2012). Ketamine effects on attention have been reported to depend on the WM load: in behavioral experiments, ketamine induced deficits were absent when the WM load was low (Harborne et al., 1996; Adler et al., 1998; Newcomer et al., 1999; Lofwall et al., 2006; Ebert et al., 2012), but present with increasing load (Adler et al., 1998; Lofwall et al., 2006; Koychev et al., 2017). A diminished task accuracy for WM tasks (Ahn et al., 2003), but not for other attention tasks (Oranje et al., 2000, 2009; Watson et al., 2009; Gunduz-Bruce et al., 2012) would fit to this pattern. The only exception from this pattern is the study of Musso et al. (2011), which showed a decrease in the hit rate in a quite simple target detection task after ketamine. This finding is somewhat surprising given that high levels of blood alcohol do not result in increased error rates in this kind of task (Colrain et al., 1993). Bearing in mind that the study of Musso et al. (2011) was conducted in an MRI scanner environment, one tentative explanation for the behavioral disruption in this easy task might be that the scanner noise functioned as potent distractor and not ketamine, but the combination of ketamine and distractor led to the behavioral impairment. In any case, in four of five studies that used a working memory-independent attention task the P3 amplitude reduction after ketamine was not accompanied by a lower task performance (Oranje et al., 2000, 2009; Watson et al., 2009; Gunduz-Bruce et al., 2012). Given this, it appears unlikely that P3b amplitude reduction after ketamine can be considered as consequence of an increased task difficulty.

Ketamine effects on the P3a were investigated in three active oddball studies (Watson et al., 2009; Gunduz-Bruce et al., 2012; Mathalon et al., 2014). In all three studies, the novel stimuli as distractors were presented in the same modality as the targets. All three reported decreased P3a amplitudes at electrodes Cz and Pz after ketamine administration. In addition, Watson et al. (2009) further reported a P3a amplitude increase at electrode Fz after ketamine relative to placebo administration, which was, however, not replicated by Gunduz-Bruce et al. (2012) and Mathalon et al. (2014). The finding of a P3a amplitude increase at Fz might have been due to pre-infusion differences between conditions, with significantly larger pre-infusion P3a amplitudes at this electrode for the placebo than ketamine session (Watson et al., 2009). Across drug-sessions, the post-infusion P3a amplitudes at Fz were smaller in the ketamine than placebo condition (Table 2 in Watson et al., 2009). In other words, the evidence for increased P3a amplitudes after ketamine in this study is at best very modest. Increased task difficulty usually results in increased P3a when distractors and targets are presented in the same modality (Table 1A). The observed decrease in P3a amplitudes after ketamine across the three studies clearly conflicts with the assumption that participants experienced the task as more difficult after ketamine.

## Discussion

The current review evaluates empirical evidence whether the ketamine-induced ERP alterations in active oddball experiments can be explained by a drug-related decreased discriminability of targets and standards, resulting in an increased task difficulty, as suggested by Schwertner et al. (2018). The major commonalities and discrepancies of ketamine and task difficulty effects on ERP components and the task accuracy are summarized in Table 3. Both ketamine and increased task difficulty are associated with decreased P3b amplitudes. However, the P3b amplitude decrease after ketamine cannot be referred to poorer task performance as the P3b amplitude reductions after ketamine were often not accompanied by a poorer task accuracy in attention tasks (Oranje et al., 2000, 2009; Watson et al., 2009; Gunduz-Bruce et al., 2012; but Musso et al., 2011). The lack of behavioral impairments in oddball and dichotic listening tasks after ketamine are in line with the assumption that the attentional impairments induced by ketamine are restricted to WM-dependent tasks, as for instance shown in the behavioral studies of Adler et al. (1998) and Lofwall et al. (2006). More importantly, not only the P3b but also the P3a amplitudes in response to distractors presented in the same modality were reduced after ketamine (Watson et al., 2009; Gunduz-Bruce et al., 2012; Mathalon et al., 2014), whereas increased task difficulty resulted exclusively in increased P3a amplitudes for such distractors (Comerchero and Polich, 1999; Demiralp et al., 2001; Polich and Comerchero, 2003; Hagen et al., 2006; Sugimoto and Katayama, 2017). Moreover, increased task difficulty leads to an increased PN (Schwent et al., 1976; Alho et al., 1992; Muller-Gass et al., 2006), whereas the PN was found to be reduced after ketamine (Oranje et al., 2000, 2009). In passive oddball experiments, increased task load in the primary task has been associated with decreased N1 amplitudes, which contrasts to ketamine studies reporting either no effects (Kreitschmann-Andermahr et al., 2001; Heekeren et al., 2008) or an increased N1 amplitude (Umbricht et al., 2000).

**Table 3:** Comparison of task difficulty and ketamine effects.

	**Increased task difficulty**	**Ketamine administration**	**Comparison**
Task accuracy in WM independent attention task	↓	Ø	≠
N1 amplitude (ignored stimuli)	↓	Ø/↑	≠
PN amplitude (attended stimuli)	↑	↓	≠
P3b amplitude to targets	↓	↓	=
P3a amplitude to distractors in the same modality	↑	↓	≠

*The table contrasts the major findings on the impact of increased task difficulty and of ketamine administration for the task accuracy in working memory independent attention task, the N1 amplitude in passive oddball paradigms, the PN as effect of attention, on the P3b to targets, and the P3a to distractors presented in the same modality as targets. The left column (“Increased task difficulty”) represents a summary of findings described in Tables 1A–D, the middle column (“Ketamine administration”) represents a summary of Table 2, and right column summarizes the contrast between the effects of ketamine administration and increased task difficulty, with “ = ” indicating similar effects, and “≠” indicating dissimilar or contrary effects*.

Overall, there is only weak empirical evidence that ketamine leads to decreased discriminability of standard and target stimuli, as suggested by Schwertner et al. (2018). The pattern of findings also argues against the assumption that the P3b amplitude reduction after ketamine could be an indirect consequence of the psychotomimetic effects of ketamine, with participants experiencing greater difficulties in performing the task after drug administration. What mechanism could provide an alternative explanation for the P3b reductions after ketamine? The most common model of the functional significance of the P3b is the context updating theory, which proposes that an incoming stimulus is compared in WM with neural representation of the previous stimulation and, in case of deviance, leads to an update of this representation (Polich, 2007). This conceptualization suggests that the P3b reflects WM-dependent functions. Thus, even though targets and non-targets are correctly classified after ketamine, the behavioral consequence in form of an update of the neural context representation might be impaired and this appears to be equally the case for targets and distractors, resulting in the reduced P3a and P3b amplitudes. Given this, the reduced P3a and P3b amplitudes might be considered as another evidence for an impairment of WM functions after ketamine.

This failure to update neural context representations might primarily be related to encoding deficits: Already in early studies, a disruption in episodic memory after ketamine has been reported (e.g., Krystal et al., 1994; Newcomer et al., 1999). Specifying the nature of this disruption, Hetem et al. (2000) and Lofwall et al. (2006) showed that in particular the encoding of new episodic information is impaired after ketamine, whereas retention and retrieval of information encoded directly before the ketamine administration remain unaffected. In an fMRI study on the effects of ketamine on spatial working memory, Driesen et al. (2013) observed a reduction of brain activation during encoding and early maintenance after ketamine, providing further support for an impairment of encoding processes by ketamine. The reduced MMN after ketamine might as well be interpreted as a deficit in forming memory traces (Umbricht et al., 2000; Schmidt et al., 2012; Rosburg and Kreitschmann-Andermahr, 2016). Of note, theoretical conceptualizations of the MMN highlight the role of the repetitive presentation of standards, with more presentations of standards leading to a more pronounced neural model and a larger MMN (Javitt et al., 1998), whereas the P3 context updating account highlights the update of the model by the target/distractor (Polich, 2007). The functional association between the MMN and P3a are still a matter of debate (e.g., Horváth et al., 2008; Rosburg et al., 2018). Further research is needed to elucidate whether the reduced P3a, P3b, and MMN after ketamine refer to disruptions of the same or different encoding processes.

In general, behavioral, neurophysiological, and neuroimaging studies on ketamine effects in healthy individuals have improved our understanding of a possible contribution of a deficient glutamatergic neurotransmission to symptoms and cognitive deficits in schizophrenia. On the basis of the NMDA receptor hypofunction hypothesis (Javitt, 2010; Kantrowitz and Javitt, 2012), new treatment strategies for schizophrenic symptoms have been stimulated, including the development of novel antipsychotic drugs. These new treatment strategies seek to improve the glutamatergic neurotransmission by ligands which bind to glycine sites of the NMDA receptor, such as d-serine and glycine, and by glycine transport inhibitors, as add-on medication to conventional anti-psychotics (Javitt, 2010; Balu and Coyle, 2015). In their meta-analysis, Cho et al. (2016) showed for example that d-serine levels were generally decreased in schizophrenia and that d-serine as add-on medication improved both positive and negative symptoms. The MMN, but to the best of our knowledge not the P3a and P3b, has been used as neurophysiological marker in some of these studies. In a recent study, Greenwood et al. (2018) showed that a 6-week treatment with glycine improved in particular negative symptoms. However, effects of glycine treatment on the MMN to duration deviants were not observed after 6 weeks of treatment, but only directly after the first glycine administration. Kantrowitz et al. (2018) observed a clinical improvement and an increase of the MMN amplitude to frequency deviants after a 6-week treatment with d-serine, with both effects being correlated. In contrast, the authors reported that treatment with the glycine transport inhibitor bitopertin had neither clinical effects nor effects on the MMN. Nevertheless, a larger randomized, double-blind phase II proof-of-concept study (without using ERPs) revealed an improvement of negative symptoms after 8 weeks of treatment with bitopertin (Umbricht et al., 2014). The sample sizes in the ERP studies of Greenwood et al. (2018) and Kantrowitz et al. (2018) were relatively small. Further research on larger patient samples is required to reveal whether clinical improvements after such novel treatments co-vary with alterations of ERP markers, like the MMN, P3a, and P3b amplitude. Moreover, these ERP markers might also be used to identify patients who might benefit from such treatment (Swerdlow et al., 2018).

## Conclusion

The conjoint reduction of the target-related P3b and the distractor-related P3a after ketamine administration conflicts with the assumption that ketamine alters the perceived salience of different categories of stimuli, as proposed by Schwertner et al. (2018). Rather, the combined reduction of the P3a and P3b suggests impaired WM updating, possibly related to general deficits in encoding of information after ketamine. Also the observation of a reduced MMN after ketamine fits well into this account (Rosburg and Kreitschmann-Andermahr, 2016). Both the P3a/P3b and the MMN might be used as neurophysiological marker for assessing the effects of drug treatments that seek to improve the glutamatergic neurotransmission.

## Author Contributions

TR searched and reviewed the literature. TR drafted the initial version of the manuscript. TR and AS were involved in the finalization of the submitted manuscript and discussed the significance of the findings.

### Conflict of Interest Statement

The authors declare that the research was conducted in the absence of any commercial or financial relationships that could be construed as a potential conflict of interest.

## Figures and Tables

**Table:** 

**ERP component**	**Description**
P3b	Also labeled “P300” and “P3;” huge centro-parietal positivity (peaking ~250–500 ms); usually investigated in active oddball experiments; cortical response to stimuli defined as targets; the P3b is presumed to reflect attention and working memory processes (Polich, 2007)
Mismatch Negativity (MMN)	Fronto-central negativity (peaking ~100–250 ms); usually investigated in passive oddball experiments; elicited by deviants; quantified by subtracting the ERPs to standard stimuli from the ERPs to deviants; the MMN is presumed to reflect pre-attentive memory-based change-detection mechanisms (Näätänen et al., 2011)
P3a	Fronto-central positivity (peaking ~250–400 ms); can be investigated in passive and active oddball experiments; elicited by deviant, rare, or novel events; reflecting an unintended attention switch (Näätänen, 1990; Polich, 2007)
Auditory P1	Also labeled, “P50;” small, early auditory evoked potential (AEP) component (peaking ~40–80 ms) with positive polarity; maximal at fronto-central electrodes; elicited by all auditory stimuli; in psychiatric research primarily investigated in the paired-click experiment (“sensory gating”); reflects sensory processes (Patterson et al., 2008)
Auditory N1	Also labeled, “N100;” large AEP component with negative polarity (peaking ~70–140 ms) following the auditory P1; maximal at fronto-central electrodes; elicited by all auditory stimuli; can be investigated among others in passive and active oddball experiments, as well as in paired-click experiment; has multiple subcomponents; reflects perceptual processes (Näätänen and Picton, 1987)
Visual P1	Early visually evoked potential (VEP) component (peaking ~100 ms); left and right temporo-parietal maxima; elicited by all visual stimuli; strongly modulated by the physical characteristics of the stimuli (Tobimatsu and Celesia, 2006)
Visual N1	VEP component with negative polarity (peaking ~140–200 ms) following the visual P1; left and right temporo-parietal maxima; has multiple subcomponents; reflects perceptual and attentional processes (Vogel and Luck, 2000); when elicited by faces, the visual N1 has also been labeled N170 (Bentin et al., 1996)
Processing negativity (PN)	Fronto-central negativity (peaking ~100–300 ms); usually investigated in dichotic listening task (attending to auditory input to one ear while ignoring the input to other ear); quantified by subtracting the ERPs to ignored stimuli from the ERPs to attended stimuli; reflects selective attention (Näätänen, 1990)
